# Morgagni Hernia: A Rare Presentation in a Young Adult

**DOI:** 10.7759/cureus.52463

**Published:** 2024-01-17

**Authors:** Kumar Sambhav, Kumar Dushyant, Shailza Raj Jayaswal

**Affiliations:** 1 Anatomy, All India Institute of Medical Sciences, Jodhpur, Jodhpur, IND; 2 Anesthesiology and Critical Care, Max Super Speciality Hospital, New Delhi, IND; 3 Radiodiagnosis, Ford Hospital and Research Center, Patna, IND

**Keywords:** omental fat, surgical repair, laparoscopic repair, congenital diaphragmatic hernia, morgagni hernia

## Abstract

Morgagni hernia is the rarest diaphragmatic hernia, occurring in only about 2% of all cases. Despite its infrequent presentation, it poses significant morbidity once the diagnosis is missed. We present a rare case of a young adult female with no predisposing factors who experienced dyspnea and retrosternal pain with unremarkable clinical findings. A posteroanterior view of the chest roentgenogram revealed a nonspecific triangular opacity at the right cardiophrenic angle. A computed tomography (CT) scan of the thorax confirmed the suspicion of a right anteromedial diaphragmatic defect with omental herniation. Exploratory laparoscopic primary repair of the hernia orifice was performed with non-absorbable sutures. CT helps in confirming the condition, and surgical repair is recommended. Morgagni hernia can present as asymptomatic or with respiratory symptoms. There is no consensus on the type of approach, but a minimally invasive approach is being preferred even in asymptomatic cases.

## Introduction

Morgagni hernia (MH) was first described in 1761 by Giovanni Battista Morgagni, regarded as the founder of pathological anatomy [1]. Anatomically, the diaphragm represents a thin, dome-shaped, musculotendinous structure separating the thoracic and abdominal cavities. Essentially, the septum transversum (ST), which is a sheet of mesoderm, eventually forms the central tendinous part, which causes this separation between cavities [2]. The diaphragm is fixed anteriorly by the small muscle fascicles along the sternal aspect of the xiphoid process. Additionally, it is reinforced by the other two anatomical attachments along the costal and lumbar extent [3]. MH results from the junction of the ST and costal elements of the diaphragm, where abdominal contents herniate into the thorax [4]. MH is the least occurring congenital diaphragmatic hernia (CDH) in only 1-5.1% of cases among the other four CDHs, namely central tendon defects of the diaphragm, Bochdalek hernia, and diaphragm eventration [5]. The first text distinctly remarks MH on the right side of the sternum and Larrey’s hernia on the left [1]. It rarely presents in adults, and its occurrence is predisposed due to conditions leading to increased intra-abdominal pressure. Mostly asymptomatic, its diagnosis can be missed routinely in an adult [5]. Thoracic roentgenogram and computed tomography (CT) thorax remain the most sensitive diagnostic modalities [5]. Ignorance of vague symptoms in young adults can lead to strangulated hernia necessitating emergency surgery [3,5]. Surgery is indicated even for asymptomatic cases. Minimally invasive surgeries are more commonly preferred nowadays for better outcomes. The laparoscopic approach employed is preferred without mesh repair in most cases, unless warranted [3,5]. In this case, we describe the clinico-radiological diagnosis and surgical intervention for a rare MH manifesting in a young adult with no evident predisposing factors.

## Case presentation

**Figure 1:**
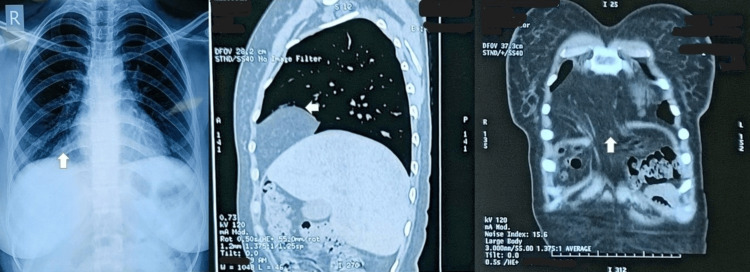
Chest roentgenogram (posteroanterior view) suggestive of nonspecific opacity at the right cardiophrenic angle (left) (white arrow). CT thorax (middle and right) confirming anteromedial diaphragmatic defect with OF herniation without bowel loops (white arrow). CT: computed tomography; OF: omental fat.

A 25-year-old normotensive, nonobese female presented with symptoms of dyspnea and retrosternal pain localized to the right side for a week. There was no history of orthopnea, chronic cough, gastrointestinal disorders, pregnancy, chronic constipation, trauma, or any other comorbidities. A chest X-ray suggested a nonspecific triangular opacity at the right cardiophrenic angle, not indicative of retrosternal mass/bowel loops, omentum, normal cardiac, lung shadows, and bronchovascular markings (Figure 1).

**Figure 2:**
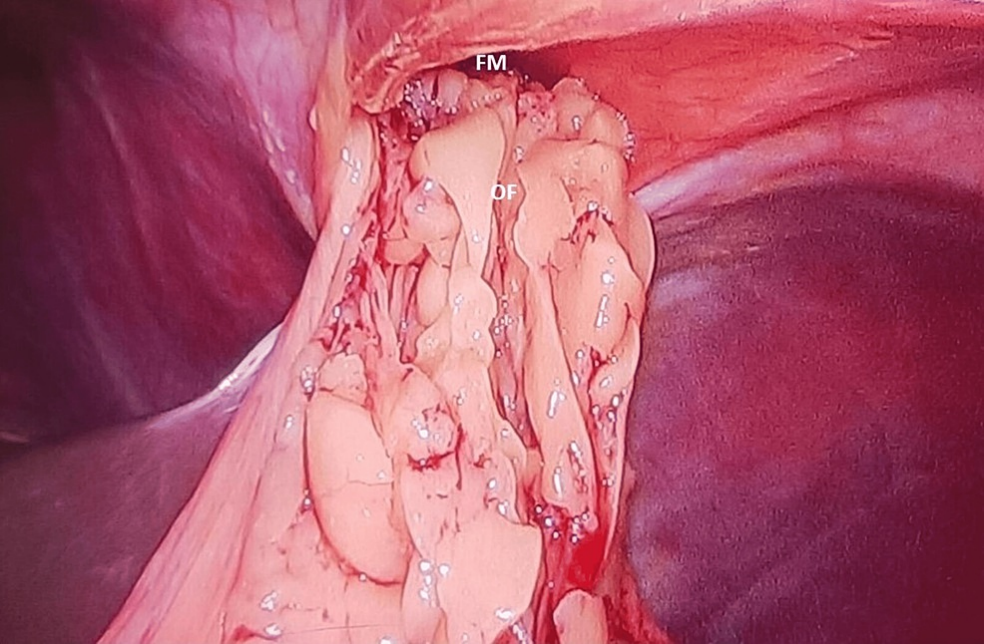
Laparoscopic real-time view of the herniating OF through the FM, measuring approximately 3 cm. OF: omental fat; FM: foramen of Morgagni.

CT thorax was ordered, given these findings that further revealed a defect measuring 30 mm (transversely) * 18 mm (anteroposteriorly) in the right anteromedial aspect of the diaphragm with herniation of the omental fat (OF) into the anteromedial aspect of the right hemithorax suggestive of MH (Figure 1). There was no radiological evidence of any bowel contents in the hernia. The patient was evaluated for exploratory laparoscopic repair of the condition. After aligning the patient in the reverse Trendelenberg maneuver, three ports were positioned to avoid proximity to the costal margin. OF with the absence of any abdominal contents was visualized herniating via the foramen of Morgagni, i.e., 3 cm, thereby confirming the pre-operative diagnosis (Figure 2).

The herniated OF was excised, and a laparoscopic primary repair of the hernia orifice was performed with a non-absorbable suture in an interrupted fashion without mesh owing to its small size and OF as its only herniated content. The patient was discharged on post-operative day 3 with minimal pain management. There was no episode of undue complications, viz., pneumothorax, and the patient’s bowel functions resumed within a few hours of surgery.

## Discussion

A young female with no evident predisposing factors presenting with retrosternal pain should always raise a high level of suspicion. As in this case, with unremarkable clinical findings, meticulous interpretation of radio imaging is paramount. A missed diagnosis can eventually lead to emergent strangulation and a consequent emergency surgical repair. CT thorax aids in confirming the diagnosis, as evident in this case. Immediate laparoscopic repair was performed for better recovery. This case report aims to reiterate the importance of clinical suspicion, radioimaging, and prompt, eventless surgical repair. MH is the least common of all CDHs usually encountered in children with respiratory complaints but it is being detected incidentally in chest radiographs of adults [6,7]. Three embryonic sources have been postulated for the development of a diaphragm, i.e., an unpaired ST, which is the first structure initially demarcating thoracic and abdominal cavities, paired pleuroperitoneal folds, and an irregular dorsal mesentery [5,8].

In the third week of gestation, ST causes separation of the pericardial region from the body and eventually realizes its typical position of the diaphragm at approximately eight weeks by growing dorsally from the ventral body wall and directing itself caudally accompanying other sources. The pleuroperitoneal folds extend medially and caudally to combine with the ST and dorsal mesentery to further complete the formation of the diaphragm at seven weeks. It is interesting to note that the right pleuroperitoneal canal closes earlier than the left. The completion of diaphragmatic structures occurs with the migration of muscle fibers from the third, fourth, and fifth cervical myotomes along with their innervation that eventually grows between the two membranes [5]. Any disturbance to the aforementioned process could result in congenital diaphragmatic hernias or congenital eventration of the hemidiaphragm. Hence, MHs occur anteriorly, possibly due to the absence of ingrowth of cervical myotomes. The sternocostal hiatus (foramen of Morgagni) is a retrosternal space arising due to the failure of fusion of fibrotendinous portions of pars tendinalis and pars sternalis, resulting in weak or incomplete muscular regions [5].

The majority of CDH (90%) occurring on the posterolateral side of the diaphragm are termed Bochdalek hernias, primarily occurring on the left side, whereas MHs occur anteriorly and more commonly on the right despite protection by the liver and pose less severe consequences [5]. These usually have a greater transverse extent than anteroposterior, as evident in this case. MH has also been documented to be more predominant in women [9].

The herniated contents in order of decreasing prevalence are fat, omentum, transverse colon, stomach, liver, small intestine, and large intestine [9]. Although the majority of cases remain asymptomatic, the presentation of symptoms can vary, with gastrointestinal complaints, vague epigastric pain, dullness, or discomfort being the only symptom in most instances. In cases of strangulation, there can be more acute symptoms. Dyspnea and palpitations are less commonly encountered [5]. In adults, the only herniated content may be omental fat without any associated conditions, in contrast to infants with MH [5]. In this case, dyspnea and retrosternal pain were the primary symptoms, along with omental fat as the only herniated content.

A chest radiograph is the first imaging modality usually done, wherein the typical finding is an opacity at the right cardiophrenic angle if the omentum is herniating with the presence of a mediastinal shift or an anteromedial mass [5,10]. A CT scan remains the most sensitive and confirmative diagnostic modality to delineate location and content and aid in ruling out differential diagnosis [5]. Contrast studies, viz. barium meal or enema, can also be employed to confirm MH [10]. MRI is employed when there is diagnostic difficulty with the fat density revealed by CT and one suspects it to be a lipoma or liposarcoma [11]. In our case, the diagnosis was confirmed through a CT scan after a suspicious right paracardiac shadow was observed on the chest radiograph. Surgery remains the gold standard treatment modality for MH [3,12]. It is prescribed even in asymptomatic cases owing to the anticipated risk of strangulation. A detailed review published in 2018 documented various surgical approaches and their implications [3].

Transthoracic and transabdominal approaches (laparotomy or laparoscopic) have been performed with an open approach in previous years, which came with longer recovery periods and increased complications in the case of laparotomies [3,7]. The transabdominal route is preferred for strangulations and bilateral and complicated hernias, whereas the thoracic approach remains widely practiced due to the fact that it provides better diaphragmatic visualization, wider exposure, and easy repairability [5,10]. There is no evidence of a common consensus as to which practice is better [3]. The laparoscopic approach was first introduced to treat MH in the mid-1900s and is now being increasingly preferred, with rising expertise, decreased hospital stay, and patient safety as the main advantages.

Besides the effectiveness of the laparoscopic technique, it can also be used to treat other conditions simultaneously and can be carried out in patients with a previous history of abdominal surgeries [13]. The utilization of mesh has been based on the size of the herniation, considering primary repair infeasible and achieving tension-free outcomes. Large-size defects with marked diaphragmatic tissue damage warrant prosthetic patch/mesh fixation. The excision of sac is contentious and dependent on many factors, such as skill, patient’s condition, cardiorespiratory complications, and pneumomediastinum, with no consequential intrathoracic adhesions. Some advocate the practice of sac removal, as doing so eliminates the possibility of future herniations [10]. Various authors have commended the minimally invasive approach, as the results are excellent with low morbidity and mortality [5,14]. In the context of this case report, it is essential to highlight the excellent results of laparoscopic repair and post-operative recovery.

## Conclusions

The diagnosis of MH necessitates a high degree of clinical suspicion in adults while assessing vague respiratory and gastrointestinal symptoms, prompting radiological investigations, followed by surgical intervention, preferably laparoscopic repair, yielding excellent results. This rare presentation of MH in a young adult would help clinicians raise suspicious levels to diagnose such cases early and help formulate a surgical guideline toward MH.
